# Prognostic Value of the Modified Glasgow Prognostic Score in Patients with Metastatic Renal Cell Carcinoma Receiving Nivolumab as Second-Line Therapy: A Multicentre Retrospective Cohort Study

**DOI:** 10.3390/medicina62091675

**Published:** 2026-08-31

**Authors:** Sedat Biter, Ertuğrul Bayram, Tolga Köşeci, Mehmet Cihan İçli, Ahmet Melih Arslan, Faruk Recep Özalp, Nadiye Sever, Ahmet Ünsal, Nargiz Majidova Altuntaş, Mustafa Seyyar, Gülhan Dinç, Mahmut Büyükşimşek, Mehmet Türker, Şendağ Yaslıkaya, Mehmet Mutlu Kıdı, Yasemin Aydınalp Camadan, Umut Kefeli, Mehmet Uzun, Hasan Çağrı Yıldırım, Hüseyin Salih Semiz, Mustafa Erman, İsmail Oğuz Kara

**Affiliations:** 1 Department of Medical Oncology, Kahramanmaraş Necip Fazıl City Hospital, Kahramanmaras 46050, Türkiye; 2 Department of Medical Oncology, Faculty of Medicine, Çukurova University, Adana 01330, Türkiye; ertugrulbayram84@gmail.com (E.B.); drtolgakoseci@gmail.com (T.K.); iokara@cu.edu.tr (İ.O.K.); 3 Department of Medical Oncology, Faculty of Medicine, Hacettepe University, Ankara 06230, Türkiye; mcihan100@hotmail.com (M.C.İ.); ermanm@hacettepe.edu.tr (M.E.); 4 Department of Medical Oncology, Faculty of Medicine, Dokuz Eylül University, Izmir 35340, Türkiye; dramarslan@gmail.com (A.M.A.); ozalpfarukrecep@gmail.com (F.R.Ö.); hsalihsemiz@hotmail.com (H.S.S.); 5 Department of Medical Oncology, Marmara University Pendik Training and Research Hospital, Istanbul 34899, Türkiye; dr.nadya@hotmail.com; 6 Department of Medical Oncology, Faculty of Medicine, Yeditepe University, Istanbul 34755, Türkiye; ahmetnsal@gmail.com; 7 Department of Medical Oncology, VM Medicalpark Maltepe Hospital, Istanbul 34846, Türkiye; nergiz.mecidova1991@gmail.com; 8 Department of Medical Oncology, Gaziantep City Hospital, Gaziantep 27470, Türkiye; mustafaseyyar27@hotmail.com; 9 Department of Medical Oncology, Hatay Training and Research Hospital, Hatay 31060, Türkiye; gulhanduyul@hotmail.com; 10 Department of Medical Oncology, University of Health Sciences, Adana City Training and Research Hospital, Adana 01260, Türkiye; mahmutbuyuksimsek@gmail.com (M.B.); mmtturker@hotmail.com (M.T.); 11 Department of Medical Oncology, Giresun Training and Research Hospital, Giresun 28200, Türkiye; drysenddag@gmail.com; 12 Department of Medical Oncology, Seyhan State Hospital, Adana 01150, Türkiye; mehmetmutlu_01@hotmail.com; 13 Department of Medical Oncology, University of Health Sciences, Şanlıurfa Mehmet Akif İnan Training and Research Hospital, Sanlıurfa 63300, Türkiye; yaseminaydinalp23@gmail.com; 14 Department of Medical Oncology, Faculty of Medicine, Kocaeli University, Kocaeli 41380, Türkiye; ukefeli@yahoo.com; 15 Department of Medical Oncology, University of Health Sciences, İzmir Tepecik Training and Research Hospital, Izmir 35180, Türkiye; memed.uzun3846@gmail.com; 16 Department of Medical Oncology, Faculty of Medicine, Ege University, Izmir 35100, Türkiye

**Keywords:** renal cell carcinoma, nivolumab, modified Glasgow Prognostic Score, C-reactive protein, albumin, systemic inflammation, prognosis, biomarker

## Abstract

*Background and Objectives*: The modified Glasgow Prognostic Score (mGPS)—a composite of serum C-reactive protein (CRP) and albumin—simultaneously reflects systemic inflammatory activity and nutritional reserve. Its specific prognostic role in patients with metastatic renal cell carcinoma (mRCC) treated with nivolumab in the second-line setting has not previously been examined in a large multicentre cohort. The aims of this study are to determine whether baseline mGPS independently predicts overall survival (OS) and progression-free survival (PFS) in this population and whether its discriminatory capacity compares favourably with the International Metastatic RCC Database Consortium (IMDC) risk score. *Methods*: This multicentre retrospective cohort study included 174 consecutive patients with histopathologically confirmed mRCC who progressed on first-line VEGF-targeted tyrosine kinase inhibitor (TKI) therapy and subsequently received nivolumab monotherapy at six oncology centres in Türkiye between January 2015 and December 2023. Baseline mGPS was calculated from serum albumin and CRP measured within four weeks before treatment initiation. Kaplan–Meier analysis with log-rank testing and Cox proportional-hazards regression were used for survival analyses; discriminatory capacity was quantified using Uno’s concordance (C) statistic. *Results*: The median follow-up was 24.2 months. The median OS was 44.5, 15.3, and 10.0 months for the mGPS 0, 1, and 2 groups, respectively (log-rank *p* < 0.0001); the median PFS was 6.7, 4.2, and 2.6 months (*p* = 0.022). On multivariable analysis, mGPS 2 independently predicted inferior OS (hazard ratio [HR] 3.61, 95% confidence interval [CI] 1.78–7.31; *p* < 0.001) and PFS (HR 1.87, 95% CI 1.17–2.97; *p* = 0.008). Uno’s C-statistic for OS was numerically higher for the combined mGPS + IMDC model (0.70) than for either the mGPS (0.66) or IMDC (0.63) alone; these differences were not formally tested and are presented descriptively. *Conclusions*: Baseline mGPS is a simple, inexpensive, and independent prognostic biomarker in mRCC patients treated with second-line nivolumab, providing discriminatory information that may be complementary alongside IMDC risk stratification. Prospective validation in randomised trials is warranted.

## 1. Introduction

Renal cell carcinoma (RCC) is the seventh most common malignancy in men and the tenth most common in women worldwide, accounting for approximately 3% of all adult cancers [1,2]. Clear cell RCC (ccRCC) constitutes the dominant histological subtype, comprising 75–80% of cases, while papillary, chromophobe, and unclassified subtypes account for the remainder [3]. Established risk factors include tobacco use, obesity, arterial hypertension, and hereditary syndromes such as Von Hippel–Lindau disease [4]. Approximately 20–30% of patients present with distant metastases at diagnosis, and a further 20–40% of those treated with curative intent eventually develop metastatic recurrence [5].

Over the past two decades, the treatment landscape for mRCC has been transformed by the sequential introduction of VEGF pathway inhibitors, mTOR inhibitors, and, most recently, immune checkpoint inhibitors (ICIs). Among ICIs, nivolumab—a fully human IgG4 monoclonal antibody targeting programmed cell death protein 1 (PD-1)—became the first approved ICI for mRCC on the basis of the phase 3 CheckMate 025 trial, in which it demonstrated superior overall survival, a higher objective response rate, and a more favourable safety profile compared with everolimus in previously treated patients [6]. Over a similar period, clinical risk-stratification tools such as the International Metastatic RCC Database Consortium (IMDC) model were developed to classify patients into prognostic groups using readily available clinical and laboratory parameters [7]. Although the treatment paradigm has since shifted toward first-line ICI-based combinations—including nivolumab plus ipilimumab and pembrolizumab plus axitinib—nivolumab monotherapy remains relevant in later-line settings and underpins several combination regimens currently in use [8].

Despite its proven efficacy, nivolumab benefits only a subset of patients, with durable responses observed in approximately 20–25% of individuals and a substantial proportion experiencing primary resistance [9]. Identifying accessible, low-cost prognostic biomarkers to guide patient counselling and treatment planning therefore remains an important clinical priority. Candidate biomarkers have included tumour mutational burden, PD-L1 expression, major histocompatibility complex alterations, T-cell infiltration patterns, and circulating immune cell subsets; however, none has yet been incorporated into routine clinical practice [10,11].

Systemic inflammation is a well-recognised driver of tumour growth, angiogenesis, and immune evasion [12]. In ccRCC specifically, constitutive secretion of interleukin-6 (IL-6), interleukin-8, and VEGF-A by tumour cells establishes a self-reinforcing immunosuppressive microenvironment [13]. Several haematological and biochemical inflammation indices—including the neutrophil-to-lymphocyte ratio, platelet-to-lymphocyte ratio, CRP, and the systemic immune-inflammation index—have shown associations with clinical outcomes in mRCC across different treatment eras [14,15].

The modified Glasgow Prognostic Score (mGPS) integrates serum CRP, a marker of systemic inflammation, with serum albumin, a marker of hepatic synthetic function and nutritional status. Originally validated by McMillan and colleagues across multiple tumour types, mGPS has subsequently demonstrated independent prognostic value in bladder cancer, upper-tract urothelial carcinoma, pancreatic cancer, colorectal cancer, and surgically managed non-metastatic RCC [16,17,18,19,20]. To date, only a single-centre Japanese series of 70 patients has examined mGPS specifically in the context of nivolumab-treated mRCC [21]. The present study was therefore designed to evaluate the independent prognostic value of baseline mGPS in the largest reported multicentre cohort of mRCC patients receiving second-line nivolumab, to compare its discriminatory capacity with the IMDC risk score, and to assess whether the two tools provide complementary prognostic information.

## 2. Materials and Methods

### 2.1. Study Design and Patient Population

This multicentre, retrospective cohort study was conducted across six oncology centres in Türkiye: Cukurova University Faculty of Medicine (Adana), Hacettepe University Faculty of Medicine (Ankara), Medikalpark Maltepe Hospital (Istanbul), Dokuz Eylül University Faculty of Medicine (Izmir), Gaziantep City Hospital (Gaziantep), and Kocaeli University Faculty of Medicine (Kocaeli). Patients were eligible if they had histopathologically confirmed RCC of any subtype, had received at least one VEGF-targeted TKI as first-line therapy for metastatic disease, had experienced documented radiological or clinical progression on that TKI, and had subsequently received nivolumab monotherapy as second-line treatment between January 2015 and December 2023. The exclusion criteria were: (1) incomplete baseline laboratory data precluding mGPS calculation; (2) receipt of systemic anticancer therapy between TKI discontinuation and nivolumab initiation; and (3) active autoimmune disease requiring systemic immunosuppression at nivolumab initiation. Five patients (2.9%) had received cabozantinib as first-line therapy; sensitivity analysis excluding these patients did not alter the primary findings. A total of 174 patients met all eligibility criteria and constituted the study population. Several investigators had moved to different institutions during the course of the study; patient-level data, however, were attributed to the treating centre at the time of nivolumab initiation, and the multicentre collaboration reflects the combined clinical experience of all participating investigators and institutions.

This study was approved by the Research Ethics Committee of Çukurova University Faculty of Medicine and was conducted in accordance with the principles of the Declaration of Helsinki. The original protocol was approved at the 147th committee meeting (decision no. 47, dated 6 September 2024); a subsequent protocol amendment extending the study to a multicentre design with the addition of collaborating investigators and institutions was approved at the 154th committee meeting (dated 18 April 2025). Owing to the retrospective, de-identified nature of the dataset, individual informed consent was waived.

### 2.2. Data Collection and Missing Data

Clinical and demographic data were extracted from institutional electronic medical records at each participating centre. Variables collected included age, sex, ECOG performance status (PS), histological subtype, sarcomatoid differentiation status, metastatic sites (lung, bone, liver, brain, lymph node), prior nephrectomy, disease presentation, first-line TKI agent, and IMDC risk category [7,22]. The IMDC risk score was calculated using clinical and laboratory parameters obtained at the initiation of second-line nivolumab, corresponding to the same baseline time point used for mGPS assessment. Baseline serum albumin (g/dL) and high-sensitivity CRP (converted to a common mg/dL reference where originally reported in mg/L) were collected within four weeks before the first nivolumab infusion. Radiographic response was assessed according to RECIST version 1.1. CRP values reported in mg/L were converted to mg/dL by dividing by 10; the prespecified threshold of >1.0 mg/dL (equivalent to >10 mg/L) was applied uniformly across all six participating centres regardless of local assay platform.

Sarcomatoid differentiation status was unavailable in 39 patients (22.4%) and lymph node involvement data in 57 patients (32.8%), reflecting heterogeneity in archived histopathological reporting across institutions and treatment years. Both variables were modelled as three-category factors (present/absent/unknown) in sensitivity analyses; the results were consistent with the primary analysis. Formal multiple imputation was not undertaken, as the parametric assumptions required could not be verified in a retrospective dataset of this size; this represents an acknowledged limitation.

Although missing data were present for sarcomatoid differentiation and lymph node involvement, these variables were retained using an explicit missing/unknown category rather than exclusion. Sensitivity analyses incorporating missingness as a separate category yielded results that were broadly consistent with the primary analysis; however, this approach should not be interpreted as equivalent to multiple imputation or as eliminating the potential for bias related to missing data.

Treating missingness as a separate category is not statistically equivalent to multiple imputation. Because missingness in sarcomatoid differentiation and lymph node status primarily reflected historical and institutional variation in histopathological reporting practice, rather than a mechanism plausibly related to the outcome itself, a missing-at-random mechanism could not be confidently assumed, and model-based multiple imputation was therefore not performed. This is acknowledged as a limitation of the present analysis: modelling missingness as a separate category can attenuate or bias covariate estimates relative to multiple imputation, and the possibility that unmeasured or informatively missing sarcomatoid and lymph node status influenced our findings cannot be excluded.

### 2.3. mGPS Calculation

The mGPS was calculated according to the validated criteria of McMillan [16]: a score of 0 was assigned when both albumin (≥3.5 g/dL) and CRP (≤1.0 mg/dL) were normal; a score of 1 when CRP was elevated (>1.0 mg/dL) with normal albumin; and a score of 2 when both hypoalbuminaemia (<3.5 g/dL) and elevated CRP were present. Isolated hypoalbuminaemia without elevated CRP was not assigned a score of 1, distinguishing mGPS from the original GPS. The pre-specified cut-offs were retained for all primary analyses; receiver operating characteristic analysis performed as a sensitivity check supported these thresholds (area under the curve for OS: CRP 0.68, albumin 0.63).

### 2.4. Outcome Definitions and Statistical Analysis

The co-primary endpoints were OS, defined as the interval from nivolumab initiation to death from any cause, and PFS, defined as the interval from nivolumab initiation to radiological/clinical progression or death, whichever occurred first. Descriptive statistics used medians with interquartile ranges (IQR) for continuous variables and frequencies with percentages for categorical variables. Kaplan–Meier curves were constructed and compared using two-tailed log-rank tests. Cox proportional-hazards regression was used for univariable (UVA) and multivariable (MVA) analysis; variables with *p* < 0.10 on UVA entered the MVA using forward conditional selection. The proportional-hazards assumption was verified using Schoenfeld residuals. The discriminatory performance of the mGPS, IMDC, and their combination was assessed using Uno’s C-statistic. Available point estimates were compared descriptively; formal pairwise statistical testing of the differences between C-statistics was not performed. Analyses were performed in SPSS version 21.0 (IBM Corp., Armonk, NY, USA); two-tailed *p* < 0.05 was considered statistically significant.

## 3. Results

### 3.1. Baseline Patient and Disease Characteristics

One hundred seventy-four patients met the eligibility criteria (Table 1 and Table 2). The median age was 61 years (IQR 54–68; range 30–88), and 72.4% were male. ECOG PS was 0 in 104 patients (59.8%), 1 in 54 (31.0%), and 2 in 16 (9.2%). Clear cell histology predominated (*n* = 149; 85.6%), with non-clear cell and unclassified tumours in 20 (11.5%) and 5 (2.9%) patients, respectively. Sarcomatoid differentiation was documented in 18 of 135 patients with available data (13.3%); de novo metastatic disease was present in 99 patients (56.9%), and nephrectomy had been performed in 138 (79.3%). The most frequent metastatic site was the lung (74.1%), followed by bone (43.7%), liver (20.1%), and brain (12.6%). By IMDC risk group, 13.2% were favourable, 66.1% intermediate, and 20.7% poor risk. First-line TKI was pazopanib in 51.1%, sunitinib in 46.0%, and cabozantinib in 2.9%. Baseline mGPS was 0 in 29.9%, 1 in 55.7%, and 2 in 14.4% of patients.

### 3.2. Comparison with Published Literature

To contextualise the present cohort relative to existing studies of inflammation-based indices in nivolumab-treated mRCC, a summary comparison is provided in Table 3. To our knowledge, the present series represents the largest cohort specifically evaluating mGPS in this clinical setting.

### 3.3. Univariable Survival Analysis

The results of the univariable Cox analysis are summarised in Table 4. An mGPS of 2 was associated with substantially higher hazards of death (HR 4.29, 95% CI 2.24–8.24; *p* < 0.001) and of progression or death (HR 1.90, 95% CI 1.20–3.01; *p* = 0.006) relative to mGPS 0. An mGPS of 1 showed a non-significant trend toward inferior OS (HR 1.82, 95% CI 0.94–3.54; *p* = 0.076). Additional variables significantly associated with shorter OS included female sex, ECOG PS ≥ 1, non-clear cell histology, sarcomatoid differentiation, absence of nephrectomy, liver/bone/brain metastases, and poor IMDC risk.

### 3.4. Multivariable Survival Analysis

A total of 102 deaths (58.6%) were observed for the OS analysis (26/52 in the mGPS 0 group, 57/97 in the mGPS 1 group, and 19/25 in the mGPS 2 group), and 148 progression-or-death events (85.1%) were observed for the PFS analysis, among the 174 patients. The final multivariable OS and PFS models each contained eight estimated covariate coefficients, corresponding to approximately 12.8 and 18.5 events per estimated coefficient, respectively. These values provide a reasonable, though not generous, event-to-parameter ratio for the models as specified. Variables with *p* < 0.10 on univariable analysis entered the multivariable model (Table 5). Baseline mGPS 2 retained independent significance for OS (HR 3.61, 95% CI 1.78–7.31; *p* < 0.001) and PFS (HR 1.87, 95% CI 1.17–2.97; *p* = 0.008). Additional independent OS predictors were female sex (HR 1.95; *p* < 0.001), non-clear cell histology (HR 1.98; *p* = 0.004), ECOG PS ≥ 1 (HR 1.87; *p* = 0.003), liver metastasis (HR 1.74; *p* = 0.018), sarcomatoid differentiation (HR 1.74; *p* = 0.031), and IMDC poor risk (HR 2.84; *p* < 0.001). The adjusted coefficient for mGPS 1 could not be reliably verified from the archived source output and is therefore not reported (Table 5); the available archived statistical output supports the reported mGPS 2 estimate, which remained unchanged.

### 3.5. Kaplan–Meier Analysis and Discriminatory Performance

**Figure 1:**
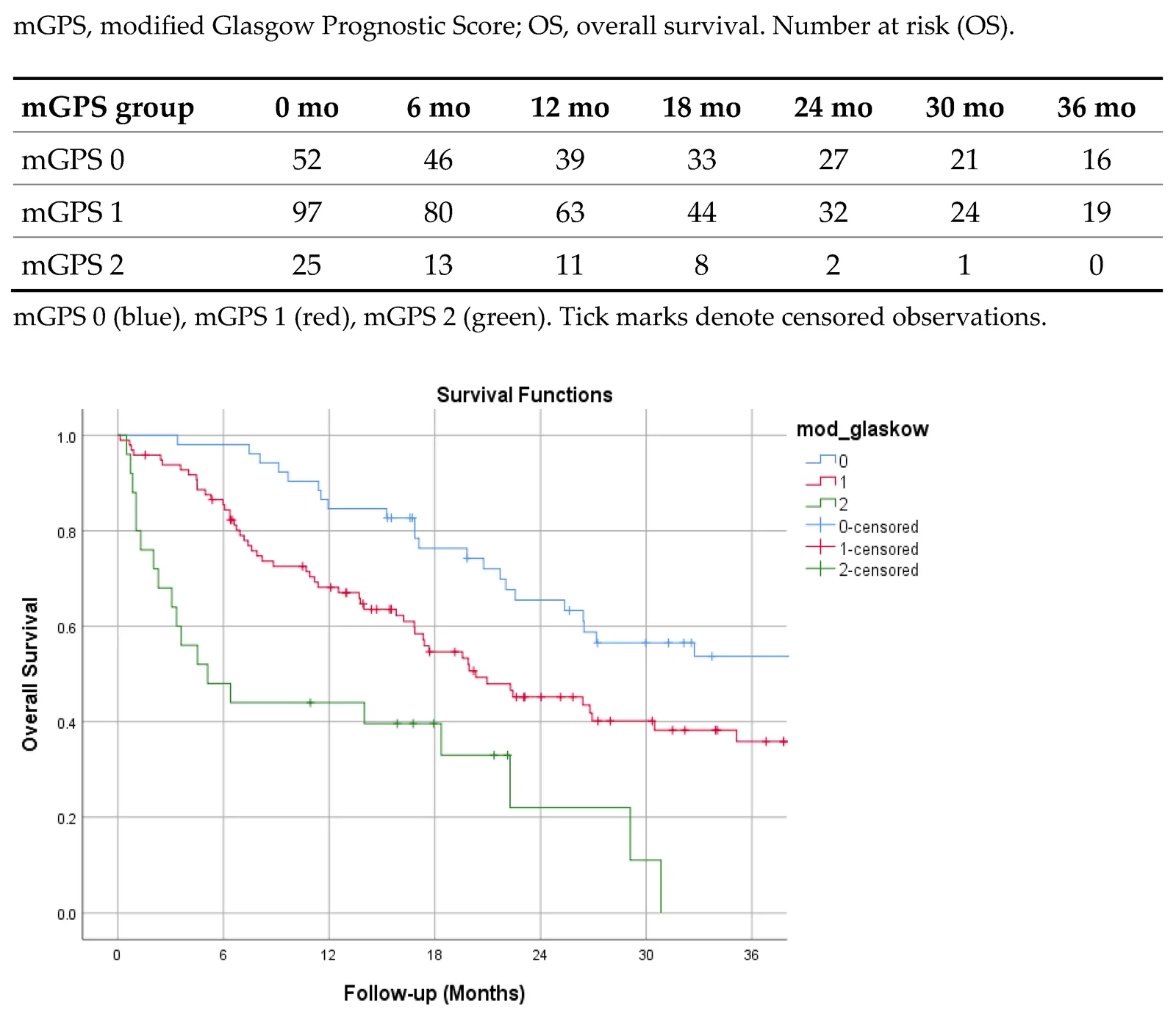
Kaplan–Meier overall survival curves stratified by baseline mGPS.

**Figure 2:**
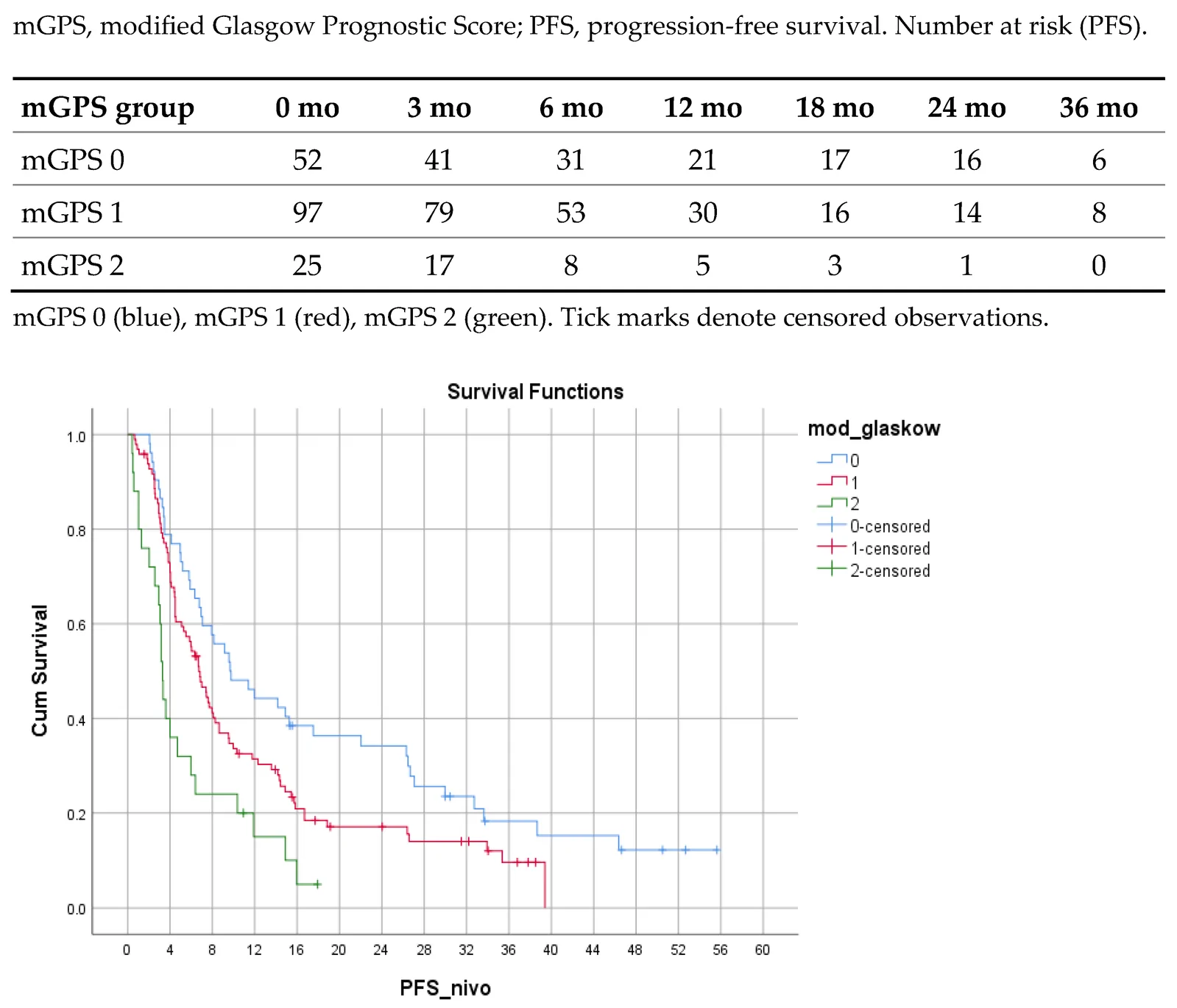
Kaplan–Meier progression-free survival curves stratified by baseline mGPS.

The median follow-up was 24.2 months. Kaplan–Meier survival outcomes according to baseline mGPS and IMDC risk groups are summarised in Table 6. The overall median OS was 20.8 months (95% CI 15.7–NE), with 12- and 24-month OS rates of 66.6% and 48.6%, respectively. The overall median PFS was 4.4 months (95% CI 3.5–6.0). Stratification by baseline mGPS produced marked separation in both endpoints: the median OS was 44.5, 15.3, and 10.0 months for mGPS 0, 1, and 2, respectively (log-rank *p* < 0.0001; Figure 1), and the median PFS was 6.7, 4.2, and 2.6 months, respectively (log-rank *p* = 0.022; Figure 2). IMDC stratification produced significant separation for OS (*p* < 0.0001) but not PFS (*p* = 0.073). The combined mGPS + IMDC model demonstrated numerically higher discrimination for OS (Uno’s C-statistic 0.70) than either the mGPS (0.66) or IMDC (0.63) alone; however, these differences were not subjected to a formal statistical comparison in the original analysis and should therefore be interpreted descriptively rather than as evidence of significantly superior discrimination.

## 4. Discussion

In this multicentre retrospective study of 174 patients with mRCC receiving second-line nivolumab, a high baseline mGPS was independently associated with shorter OS and PFS, even after adjustment for IMDC risk group, ECOG PS, histological subtype, and sarcomatoid differentiation. To our knowledge, this represents the largest published cohort examining mGPS specifically in the nivolumab treatment context and the first to compare its discriminatory capacity with the IMDC risk score using Uno’s C-statistic.

Several biological mechanisms may explain the association between elevated mGPS and poorer outcomes in patients receiving PD-1 blockade. CRP is synthesised by hepatocytes in response to pro-inflammatory cytokines, principally IL-6 and IL-1β [23]. In ccRCC, KIM-1/HAVCR1 expression activates IL-6/JAK/STAT-3 signalling, driving autocrine tumour proliferation, apoptosis resistance, and VEGF-mediated angiogenesis [13,24]. Critically, the same cytokines that stimulate hepatic CRP production also expand myeloid-derived suppressor cells and regulatory T cells, generating an immunosuppressive microenvironment that directly antagonises the antitumour immune response elicited by anti-PD-1 therapy [25]. Elevated baseline CRP may therefore reflect not merely tumour burden but also the degree of immunological dysfunction that nivolumab must overcome, offering a plausible mechanistic explanation for the reduced benefit observed in mGPS 2 patients.

An important question is whether mGPS reflects overall disease prognosis irrespective of treatment or whether it may also be associated with response to nivolumab specifically. The absence of a contemporaneous comparator arm in this study precludes definitive resolution of this distinction; addressing it would require a randomised or propensity-matched design. Nonetheless, the mechanistic link between IL-6-driven immunosuppression and attenuated checkpoint-inhibitor efficacy provides biological plausibility for a treatment-specific contribution beyond a simple performance status surrogate effect.

The albumin component of the mGPS contributes complementary prognostic information. Hypoalbuminaemia reflects cachexia-related reduced protein intake, IL-6-mediated suppression of hepatic albumin synthesis, and inflammation-related vascular hyperpermeability [26]. A meta-analysis of 14 studies and 8062 patients with RCC found that lower pretreatment albumin independently predicted worse OS (pooled HR 2.14, 95% CI 1.69–2.71) [27]. Hypoalbuminaemia is also associated with impaired T-cell function, a process directly relevant to the anti-PD-1 mechanism of action [28]. The integration of CRP and albumin within the mGPS therefore captures two converging pathophysiological axes that may independently undermine nivolumab efficacy.

Our findings are consistent with a meta-analysis of 4889 patients with RCC demonstrating that elevated CRP independently predicts worse OS across treatment modalities [29,30]. Our findings support previous reports regarding the prognostic value of systemic inflammation and suggest that the mGPS may provide additional information beyond conventional IMDC risk stratification in patients treated with second-line nivolumab. The combined mGPS + IMDC model showed a numerically higher Uno’s C-statistic than either score alone (0.70 vs. 0.66 for mGPS and 0.63 for IMDC); because this difference was not formally tested, it should be regarded as descriptive and hypothesis-generating rather than as evidence of a true incremental gain [7,22].

Because the difference in Uno’s C-statistic was not subjected to a formal statistical comparison, we are cautious about over-interpreting it; nevertheless, the observed numerical pattern raises the possibility that the mGPS captures a prognostic dimension—the host inflammatory-nutritional phenotype—that is not fully represented by the conventional IMDC variables. While the IMDC primarily reflects tumour burden and clinical status, the mGPS additionally incorporates the host inflammatory and nutritional response, and prospective studies with formal model-comparison procedures are required to determine whether this represents a statistically and clinically meaningful improvement in discrimination.

From a practical perspective, the mGPS offers several advantages over emerging molecular and immunological biomarkers. Both CRP and albumin are inexpensive, routinely available laboratory parameters that can be measured in virtually all oncology centres without additional cost or specialised infrastructure. Therefore, incorporation of the mGPS into routine clinical assessment may be particularly valuable in resource-limited settings where access to advanced genomic or immunological testing remains restricted. The simplicity and reproducibility of the mGPS further support its potential role as a complementary prognostic tool alongside established risk models such as the IMDC.

Importantly, a high mGPS should not in itself be interpreted as a reason to withhold nivolumab, particularly given the absence of a comparator arm in this study. Rather, the mGPS may assist clinicians in identifying patients with a particularly adverse prognosis who may benefit from closer clinical monitoring, early optimisation of nutritional and supportive care, proactive symptom management, and timely integration of palliative care. In prospective trials, the mGPS may also serve as a pragmatic stratification factor or enrichment biomarker for studies evaluating intensified or combination treatment strategies in patients with a high inflammatory-nutritional burden.

The independent association of female sex with inferior OS (HR 1.95; *p* < 0.001) merits comment, as this is not captured in standard risk scores. Pooled analyses of ICI trials have reported heterogeneous sex-based differences in efficacy across tumour types [31,32], potentially reflecting differences in baseline immune activation, sex hormone-mediated immunomodulation, and PD-L1 expression patterns. While this finding may partly reflect residual confounding in a retrospective analysis, its biological plausibility justifies its acknowledgement as a clinically relevant observation warranting prospective evaluation. Nevertheless, this observation should be interpreted cautiously. The relatively smaller number of female patients and the retrospective nature of the study may have contributed to residual confounding. Consequently, this finding should be considered hypothesis-generating and requires confirmation in larger prospective cohorts.

Our study substantially extends the work of Fujiwara et al. [21], the only prior study examining the mGPS in nivolumab-treated mRCC, through a 2.5-fold larger sample, a multicentre design spanning six institutions across different regions of Türkiye, comparison with the IMDC using Uno’s C-statistic, and comprehensive multivariable adjustment for histological subtype, sarcomatoid differentiation, and all major metastatic sites.

The contemporary first-line mRCC treatment landscape is increasingly dominated by ICI-ICI and ICI-VEGF-TKI combination regimens, and the exact magnitude of the associations observed in this historical second-line nivolumab cohort therefore cannot be directly extrapolated to current first-line practice. Nevertheless, systemic inflammatory and nutritional status represent treatment-independent dimensions of host biology that are conceptually applicable across treatment settings, supporting the rationale for evaluating the mGPS prospectively in contemporary ICI-based combination cohorts.

This study has several limitations that should be considered when interpreting the results. First, the retrospective design carries risks of selection bias and incomplete covariate ascertainment, notably for sarcomatoid differentiation (missing in 22.4%) and lymph node status (missing in 32.8%); sensitivity analyses did not materially alter the primary findings, though formal multiple imputation was not performed. Second, CRP and albumin assays across six institutions may have used heterogeneous analytical platforms, introducing measurement variability. Third, pretreatment CRP and albumin may have been influenced by prior TKI therapy, complicating interpretation of the baseline mGPS as a pure reflection of tumour biology. Fourth, all patients received nivolumab strictly in the second-line setting prior to widespread adoption of first-line ICI-based combinations; whether the mGPS retains prognostic significance in other treatment contexts remains to be established. Finally, the absence of a comparator arm precludes distinguishing a general prognostic from a nivolumab-specific predictive role for the mGPS.

## 5. Conclusions

In this multicentre retrospective cohort, baseline mGPS was independently associated with both overall survival and progression-free survival in patients with mRCC receiving second-line nivolumab. An mGPS of 2 was associated with an approximately 3.6-fold higher hazard of death and a 1.9-fold higher hazard of progression or death after multivariable adjustment for IMDC risk group and other established prognostic factors. The combined mGPS + IMDC model showed a numerically higher Uno’s C-statistic than either score alone; however, this difference was not formally tested and should therefore be regarded as descriptive rather than as evidence of a proven incremental gain in discrimination. Given its simplicity, low cost, and broad availability, mGPS may represent a practical adjunct to established prognostic assessment in this setting; its clinical utility, and any role in guiding treatment decisions, require prospective validation in contemporary ICI-based treatment cohorts before they can be established.

## Figures and Tables

**Table 1:** Baseline patient and disease characteristics, stratified by baseline mGPS group (*n* = 174).

Characteristic	Overall (*n* = 174)	mGPS 0 (*n* = 52)	mGPS 1 (*n* = 97)	mGPS 2 (*n* = 25)	*p*
Age, median (IQR), years	61 (54–68)	60	63	62	0.194
Female sex, *n* (%)	48 (27.6)	21 (40.4)	20 (20.6)	7 (28.0)	0.037
ECOG PS ≥ 1, *n* (%)	70 (40.2)	15 (28.8)	40 (41.2)	15 (60.0)	0.032
Non-clear cell histology, *n* (%)	25 (14.4)	10 (19.2)	13 (13.4)	2 (8.0)	0.387
Sarcomatoid differentiation, *n* (%) ^a^					0.064
Present	18 (10.3)	3 (5.8)	12 (12.4)	3 (12.0)	
Absent	117 (67.2)	44 (84.6)	64 (66.0)	9 (36.0)	
Unknown	39 (22.4)	5 (9.6)	21 (21.6)	13 (52.0)	
De novo metastatic, *n* (%)	99 (56.9)	26 (50.0)	53 (54.6)	20 (80.0)	0.036
Nephrectomy, *n* (%)	138 (79.3)	47 (90.4)	77 (79.4)	14 (56.0)	0.002
Lung metastasis, *n* (%)	129 (74.1)	34 (65.4)	78 (80.4)	17 (68.0)	0.102
Bone metastasis, *n* (%)	76 (43.7)	21 (40.4)	43 (44.3)	12 (48.0)	0.804
Liver metastasis, *n* (%)	35 (20.1)	12 (23.1)	18 (18.6)	5 (20.0)	0.806
Brain metastasis, *n* (%)	22 (12.6)	5 (9.6)	14 (14.4)	3 (12.0)	0.697
IMDC risk group, *n* (%)					<0.001
Favourable	23 (13.2)	13 (25.0)	10 (10.3)	0 (0.0)	
Intermediate	115 (66.1)	34 (65.4)	67 (69.1)	14 (56.0)	
Poor	36 (20.7)	5 (9.6)	20 (20.6)	11 (44.0)	
First-line TKI, *n* (%)					0.263
Sunitinib	80 (46.0)	22 (42.3)	47 (48.5)	11 (44.0)	
Pazopanib	89 (51.1)	30 (57.7)	45 (46.4)	14 (56.0)	
Cabozantinib	5 (2.9)	0 (0.0)	5 (5.2)	0 (0.0)	

Abbreviations: mGPS, modified Glasgow Prognostic Score; IQR, interquartile range; ECOG PS, Eastern Cooperative Oncology Group Performance Status; IMDC, International Metastatic Renal Cell Carcinoma Database Consortium; TKI, tyrosine kinase inhibitor. Categorical variables were compared with the χ^2^ test (or Fisher’s exact test where any expected cell count was <5); age was compared with the Kruskal–Wallis test. ^a^ Sarcomatoid differentiation status was unavailable in 39 patients (22.4%) and is shown as a separate ‘Unknown’ category; the χ^2^ test for this variable (*p* = 0.064) treats ‘Unknown’ as a third category.

**Table 2:** Best radiographic response to nivolumab by baseline mGPS group.

Response	mGPS 0 (*n* = 51) ^a^	mGPS 1 (*n* = 92) ^a^	mGPS 2 (*n* = 24) ^a^	*p*
CR, *n* (%)	5 (9.8)	1 (1.1)	0 (0.0)	
PR, *n* (%)	12 (23.5)	35 (38.0)	6 (25.0)	
SD, *n* (%)	6 (11.8)	18 (19.6)	4 (16.7)	
PD, *n* (%)	28 (54.9)	38 (41.3)	14 (58.3)	
ORR (CR + PR), *n* (%)	17 (33.3)	36 (39.1)	6 (25.0)	0.408
DCR (CR + PR + SD), *n* (%)	23 (45.1)	54 (58.7)	10 (41.7)	0.161

^a^ Denominators reflect patients with a documented RECIST 1.1 response assessment (167/174, 96.0%); 7 patients were not radiographically evaluable and are excluded from this table. mGPS, modified Glasgow Prognostic Score; RECIST, Response Evaluation Criteria in Solid Tumors; CR, complete response; PR, partial response; SD, stable disease; PD, progressive disease; ORR, objective response rate; DCR, disease control rate. ORR and DCR did not differ significantly across mGPS groups and did not decline monotonically with increasing mGPS; these findings do not support a clear association between baseline mGPS and objective tumour response to nivolumab and are consistent with interpreting mGPS primarily as a prognostic biomarker in this single-arm cohort. A definitive predictive-versus-prognostic distinction would require a comparator arm or a treatment-by-biomarker interaction test, which this single-arm design cannot provide.

**Table 3:** Comparison with published studies evaluating inflammation-based indices in nivolumab-treated mRCC.

Study	Design	*n*	Setting	mOS, mo	Key Finding
Fujiwara et al. (2021) [21]	Retrospective, single-centre	70	Nivolumab 2L	NR	mGPS predicts OS/PFS
De Giorgi et al. (2019) [14]	Retrospective, multicentre	389	Nivolumab 2L	NR	SII + BMI predict OS
Present study	Retrospective, 6 centres	174	Nivolumab 2L	20.8	mGPS predicts OS/PFS; complementary to IMDC

2L, second-line; SII, systemic immune-inflammation index; BMI, body mass index; NR, not reported; mOS, median overall survival; mGPS, modified Glasgow Prognostic Score; OS, overall survival; PFS, progression-free survival; IMDC, International Metastatic Renal Cell Carcinoma Database Consortium.

**Table 4:** Univariable Cox proportional-hazards analysis for OS and PFS.

Variable	OS: HR (95% CI); *p*	PFS: HR (95% CI); *p*
mGPS (ref = 0)	—	—
*mGPS 1*	1.82 (0.94–3.54); 0.076	1.45 (0.96–2.19); 0.078
*mGPS 2*	4.29 (2.24–8.24); <0.001	1.90 (1.20–3.01); 0.006
Sex (female vs. male)	1.82 (1.23–2.68); 0.003	1.38 (0.98–1.96); 0.069
ECOG PS (≥1 vs. 0)	2.14 (1.45–3.15); <0.001	1.61 (1.14–2.28); 0.007
Histology (non-ccRCC vs. ccRCC)	1.89 (1.19–3.01); 0.007	1.52 (0.99–2.33); 0.055
De novo metastatic disease	1.34 (0.91–1.97); 0.138	1.22 (0.86–1.72); 0.264
Sarcomatoid differentiation	2.01 (1.23–3.28); 0.005	1.64 (1.03–2.62); 0.037
Nephrectomy (no vs. yes)	1.77 (1.14–2.75); 0.011	1.41 (0.94–2.12); 0.097
Liver metastasis	1.98 (1.28–3.06); 0.002	1.57 (1.04–2.36); 0.031
Bone metastasis	1.67 (1.14–2.44); 0.009	1.43 (1.01–2.02); 0.044
Brain metastasis	1.89 (1.13–3.16); 0.015	1.44 (0.88–2.34); 0.147
IMDC Intermediate (vs. Favourable)	2.24 (1.12–4.47); 0.022	1.56 (0.85–2.86); 0.151
IMDC Poor (vs. Favourable)	4.87 (2.36–10.05); <0.001	2.34 (1.22–4.49); 0.010

HR, hazard ratio; CI, confidence interval; ccRCC, clear cell renal cell carcinoma; OS, overall survival; PFS, progression-free survival; mGPS, modified Glasgow Prognostic Score; ECOG PS, Eastern Cooperative Oncology Group Performance Status; IMDC, International Metastatic Renal Cell Carcinoma Database Consortium.

**Table 5:** Multivariable Cox proportional-hazards analysis: independent predictors of OS and PFS.

Variable	OS: HR (95% CI); *p*	PFS: HR (95% CI); *p*
mGPS (ref = 0)	—	—
*mGPS 1*	NR	NR
*mGPS 2*	3.61 (1.78–7.31); <0.001	1.87 (1.17–2.97); 0.008
Sex (female vs. male)	1.95 (1.34–2.85); <0.001	1.44 (1.01–2.05); 0.044
ECOG PS (≥1 vs. 0)	1.87 (1.24–2.82); 0.003	1.52 (1.06–2.17); 0.022
Histology (non-ccRCC vs. ccRCC)	1.98 (1.24–3.14); 0.004	1.60 (1.03–2.49); 0.037
Sarcomatoid differentiation	1.74 (1.05–2.89); 0.031	1.53 (0.95–2.47); 0.078
Liver metastasis	1.74 (1.10–2.76); 0.018	1.48 (0.97–2.26); 0.072
IMDC Poor vs. Favourable	2.84 (1.68–4.80); <0.001	1.90 (1.19–3.03); 0.007

Abbreviations: OS, overall survival; PFS, progression-free survival; HR, hazard ratio; CI, confidence interval; mGPS, modified Glasgow Prognostic Score; ECOG PS, Eastern Cooperative Oncology Group Performance Status; ccRCC, clear cell renal cell carcinoma; IMDC, International Metastatic Renal Cell Carcinoma Database Consortium. Variables with *p* < 0.10 on univariable analysis were entered using forward conditional selection. Schoenfeld residuals confirmed the proportional-hazards assumption for all covariates. NR, not reported; the archived source output did not permit reliable verification of the adjusted mGPS 1 coefficient. To avoid reporting an unverified estimate, this value was omitted; the available archived statistical output supports the reported mGPS 2 estimate, which is unaffected.

**Table 6:** Kaplan–Meier survival outcomes by mGPS and IMDC risk group.

Subgroup	*n*	Median OS, mo (95% CI)	Median PFS, mo (95% CI)
Overall cohort	174	20.8 (15.7–NE)	4.4 (3.5–6.0)
mGPS 0	52	44.5 (27.3–NE)	6.7 (3.6–13.1)
mGPS 1	97	15.3 (11.0–24.2)	4.2 (2.9–6.2)
mGPS 2	25	10.0 (4.6–17.5)	2.6 (2.0–5.6)
Log-rank *p*	—	*p* < 0.0001	*p* = 0.0216
IMDC Favourable	23	NE	8.1 (4.3–NE)
IMDC Intermediate	115	19.4 (14.1–26.3)	4.2 (3.1–5.9)
IMDC Poor	36	9.1 (6.2–14.0)	2.4 (1.8–4.0)
Log-rank *p*	—	*p* < 0.0001	*p* = 0.0728

Abbreviations: mGPS, modified Glasgow Prognostic Score; IMDC, International Metastatic Renal Cell Carcinoma Database Consortium; OS, overall survival; PFS, progression-free survival; CI, confidence interval; NE, not evaluable; mo, months. Log-rank *p*-values compare all three strata simultaneously.

## Data Availability

The datasets generated and/or analysed during the current study are not publicly available because of institutional data-sharing and data-protection restrictions. De-identified data may be made available from the corresponding author upon reasonable request, subject to institutional approval and availability of the archived source data.

## Abbreviations

CI	confidence interval
CRP	C-reactive protein
ECOG PS	Eastern Cooperative Oncology Group performance status
HR	hazard ratio
ICI	immune checkpoint inhibitor
IMDC	International Metastatic RCC Database Consortium
mGPS	modified Glasgow Prognostic Score
mRCC	metastatic renal cell carcinoma
OS	overall survival
PD-1	programmed cell death protein 1
PFS	progression-free survival
RCC	renal cell carcinoma
TKI	tyrosine kinase inhibitor
